# Book Reviews

**Published:** 1815-10

**Authors:** 


### CRITICAL ANALYSIS
### OF RECENT PUBLICATIONS
IN THE
### DIFFERENT BRANCHES OF PHYSIC, SURGERY, AND MEDICAL PHILOSOPHY.

*Case of a Fœtus found in the Abdomen of a Young Man, at Sherborne, in Dorsetshire,* by NATHANIEL HIGHMORE, Surgeon, and Member of the Royal College of Surgeons, in London. With explanatory Engravings. 4to. Longman, &c. London. pp. 30.

THE diligence of modern practitioners in the examination of the bodies of those who die of peculiar complaints, has very much enriched our knowledge of medicine. Morbid anatomy, in itself, is a very insufficient direction in the inquiry concerning the causes and seat of diseases. Without a previous history of the symptoms, we gain but little by seeing what has been their effects.

The present history does great honour to Mr. Highmore, for his early discovery of the anomalous nature of the complaint, for the attention with which he watched its progress and traced its history, and certainly not least for the diligence by which he acquired an opportunity of examining the body in its most recent condition. The subject is so generally known, that we shall only, in as few words as possible, describe an event which must be seen again and again before we can be perfectly satisfied of the whole progress.

Mr. Highmore first saw the patient in May, 1814. He was at that time so dangerously ill, and so much swollen, that his mother was fearful he would burst. Mr. H. then learned, that in the preceding January he had been seized with rigor, and consequent fever, from which he had never perfectly recovered. During his illness, he suffered much from diarrhœa, and was gradually reduced to a condition which rendered him incapable of quitting his bed. On being interrogated concerning his feelings, he complained of pain in his body, which was extremely swollen. The abdomen, on examination, exhibited every appearance of ascites. But, besides the fluctuation which was evident, there was found, immediately over the splenic region, a projection which the eye might detect, and which, on the slightest pressure, produced an increase of pain.

"I was (says Mr. Highmore) forcibly struck with the violence of of its pulsation, so much so, that, if it had been differently situated, I should have considered it an *aneurism* of considerable magnitude.

"Independently, however, of the pulsatory feel, there was a different kind of motion perceptible, as if the part were affected by spasmodic action: a circumstance for which I could not satisfactorily account; and which appeared to me still more inexplicable, from the following statement of the lad's mother. She observed, that a few days previous to that time, he exclaimed, affrighted, '*Mother! do come to me, I have something* ALIVE *in my body!*' Upon saying which, he almost immediately fainted. She went to him, and found a very considerable motion in the swelling, which was not merely apparent to the touch, but equally visible to the eye; and resembling, as she would have expressed herself, the motion of a child during gestation. This, according to the mother's account, was the *first* time he had ever complained of, or felt, any *motion* in the part; as will presently appear by the statement she subsequently gave me, respecting his general state of health, from the time of his birth to that of my seeing him."

Mr. Highmore, it will readily be presumed, conceived the disease in the spleen. His prognosis to the mother was unfavourable; and, from a conviction of some peculiarity in the case, he took this first opportunity to procure the promise of an examination after death. Mercury, with squills, produced some remission of the symptoms; and opium relieved the pain. In this manner things continued for exactly thirty-one days.

"I was unavoidably prevented from seeing him for two days, until Thursday, June 9th, his mother having previously called upon me, to say that he was much worse than when I last saw him. She also stated, that he had 'vomited a considerable quantity of thin and clotted blood;' that he was 'exceedingly faint,' and could scarcely breathe, seemingly from excessive pain. I desired her to preserve whatever might pass from him until I could see him again in the evening.

"I indeed found him in a state of great suffering, which he principally referred to his back; his body was covered with a profuse perspiration, and his pulse was greatly accelerated; while at the same time he was voiding, per anum, long portions of firmly coagulated blood, one piece of which, that was partly drawn from him, would have measured more than two feet in length: it was elastic, and, when expelled, bore the perfect impression of the intestinal convolutions.* He was at this time perfectly sensible, and intreated me to send him another pill (meaning the opiate pill), which I promised him I would do. I then intimated to his friends, that he was sinking so rapidly as scarcely to leave a chance of his surviving the night: at all events, I directed them carefully to mark

"* In the course of this day he evacuated, from the stomach and anus, above a gallon of fluid and coagulated blood."

every every symptom distinctly, and to report them to me early on the following morning."

Mr. Highmore's prognosis proved correct, the youth dying the same night in great agonies.

On examining the body, a large tumour presented itself as soon as the parieties of the abdomen were divided, and the viscera exposed.

"It occupied portions of the epigastric, umbilical, and left hypochondriac regions; and was uncovered by the omentum, which was found in a ruffled state, lying above the tumour.

"In tracing the course of the intestines upwards, and examining more particularly the tumour, I discovered that the jejunum was continued into the anterior and sinister-lateral-inferior part of the sac, inclosing the substance, of which, by its apparent expansion, it evidently formed a part. I then traced the course of the duodenum from the pyloric orifice of the stomach, and observed that its whole curve was firmly attached to and connected with the sac, and that this intestine, also terminating in it, formed the anterior and dexter-lateral portion."

The spleen appeared compressed, and only slightly inflamed. Notwithstanding the intermissions in the pulse which occurred in the early part of Mr. H.'s attendance, no other mischief appeared in the thorax than that the pericardium was distended, and contained seven ounces of a serous fluid.

"As this tumour," continues the account, "appeared to be the only diseased part, and was evidently contained within the intestine, with a free communication between it, the duodenum, and the jejunum, I proceeded to remove it from the body; and, in order to prevent any alimentary, or other matter, from escaping, I secured the jejunum, below the tumour, with two ligatures, and divided it between them; but, as the stomach was so closely connected with the tumour above, I considered that one ligature would there be sufficient, which I applied round the pyloric orifice, and divided the neck of the stomach. The only attachments that now remained were to the mesentery, which had confined it close to the spine, and to the pancreas. I divided the mesentery; but, as the pancreas so strongly adhered to it, immediately over the vertebræ, a portion of it was removed with the tumour.

"Having sewed up the body, I conveyed the tumour to my house, and found its weight to be four pounds and half; and, whilst in the act of placing it in a vessel, where it was to remain during the night, my fore-finger accidentally slipped round the curve of the arm, at the elbow-joint, which first gave me the idea of its partaking an animal form. At this circumstance I felt greatly astonished; and the more so, when, on my endeavouring to trace, through the sac, the extremity of the limb, I could count five digitals!

"The

"The discovery thus made gave rise to a variety of conjectures, and speculative arguments; and in order to ascertain that the youth from whom the fœtus had been removed was really a male person, I accompanied these gentlemen to the mother of the deceased's house, where they each respectively examined the body, and became fully satisfied that he was a perfectly formed male; a circumstance on which some doubts had been expressed."

It will easily be supposed that the report quickly spread all over the country, so as to be extremely troublesome to the surgeon, who very prudently brought his subject to London, where it soon found its way to the College of Surgeons, at which place the writer of this article saw it.

The following is the description of the fœtus, and of the sac or cyst in which it was contained.

"On opening the sac by an oblique incision, through the anterior and thinnest part, an imperfect human fœtus was discovered. It lay on its left side, with the right arm, which was perfect, bent down upon the right hip; the left, which was very imperfectly formed, was resting against the leg, the thigh of which was bent up towards the abdomen; and the leg was bent at an acute angle upon the thigh.

"The fœtus was connected with the sac by a short thick funis which arose from its abdomen, rather towards the left side, passed forwards between the left arm and leg, and entered the sac at the posterior and upper part. It had no head; but at the basis of a denuded first vertebræ some slips of skin arose, which followed nearly the course of the funis, with some medullary substance, around which was entangled a considerable quantity of matted hair, part of which measured twelve inches in length. There was also adhering to this skin a thin piece of bone, appearing to be a portion of cranium. The spine was much curved. There were two superior extremities, the right of which was perfectly formed, but had been fractured just above the carpal bones: the upper end of the radius was also dislocated, and protruded through the integuments. The left upper extremity was less perfectly formed; it was short, ill-shaped, and had only three fingers, with very long nails. The scapula of this arm rested on the side, almost as low down as the hip.

"There was only one inferior extremity: the thigh was bent forwards upon the body of the child, and the leg bent, sharp-angle, upon the thigh; the skin being common to each almost half their length, forming a kind of web, uniting the two together. The knee was dislocated; the skin over it had been absorbed, and the joint was exposed. The ankle was also dislocated, and turned inwards; the common integuments had been absorbed, and the bones were exposed and perishing. It had six imperfectly-formed toes.

"The left inferior extremity was wanting; but there was a considerable surface divested of skin at the spot where it should have been, been. This surface was regular and smooth, without any projecting bone, excepting at the inferior part, where a portion of ischium protruded.

"There was a quantity of sebaceous matter between the extremities and the body of the fœtus: on the upper part of the thorax was a long fleshy excrescence, somewhat like the papilla of an elderly woman; and the appearance of the genitals favoured the idea of the fœtus being a female.

"The sac which contained the fœtus was made up of two distinct portions: the larger portion, which was thick, spongy, and highly vascular, involved the greater part of the fœtus. A small portion of the buttocks, the bent carpus, and the right arm, together with the foot and ankle, were covered by, and lying in contact with, the inner surface of the intestine of the boy, which formed the second portion of the sac.

"The opening into the intestine seems to have been partly into the duodenum, and partly into the jejunum. The duodenum entered the cyst, the posterior part having been ruptured, while the anterior was continued into the jejunum, and formed the small portion of the described cyst. The jejunum emerged out of the sac, with a somewhat similar orifice, and was not far removed from the orifice of the duodenum; so that it was only the anterior portion of the intestinal canal that was dilated: the muscular fibres of the intestine could be easily traced, and were lost in the thick and vascular portion.

"On the outside of the sac, near the place where the funis entered, a great number of large vessels were observable; and on the inside of the sac, not far from the insertion of the funis, was a large arterial branch ruptured, from whence the hæmorrhage proceeded, which was the immediate cause of the lad's death."

This description, though, for the most part, very perspicuous, is much illustrated by two very well executed engravings; but we do not clearly comprehend whether the rupture in the posterior part of the duodenum happened after death, or occurred during life. The hæmorrhage is accounted for by the rupture of a considerable branch of the mesenteric artery.

A history of the boy from his birth follows, of which we shall extract an epitome.

1793. At the end of this year he was born, at Hilfield, in Dorset; was wet-nursed, and seemed healthy till the seventh year of his age.

1805. His mother took him to the lady of the late Admiral Digby, the Lady Bountiful of Mintern-Magna, on account of pains in his bowels. He took vermifuge medicines, and recovered slowly, without voiding worms. His belly was so swollen during his illness, that he could not button his clothes. On his recovery, his clothes could be buttoned;

 but but from that time, and not before, as it appears by the account, there was always a visible enlargement.

1806. During this year he was well during the day, but often restless, and sometimes delirious during the night, never making any complaints but of his belly. His state of health was generally good till

1811. In March he had severe pain and swelling in his abdomen, so that he was unable to preserve the erect posture. From these he soon recovered, and seemed better than at any period of his whole life, till April,

1812, when he was suddenly seized with extreme pain in his back and loins. From these he soon recovered sufficiently to resume, but by no means in a fit state to undertake, his employment of a carter's boy.

1814, occurred the exposure to the snow before-mentioned, and all the other events related at the beginning of the paper.

The following are Mr. Highmore's reflections.

"Amongst professional men, who have examined this singular fœtus, a variety of opinions and conjectures have been formed, some of which it may be well to notice. And first, with regard to its relation to the intestine, a question arises, whether or not it was originally deposited there.

"I am inclined to think, that the weight of evidence is in favour of its not having been placed first in that part. The sac, as already mentioned, was formed of two distinct portions. The portion highest in the body was thick, highly vascular, and involved the greatest part of the fœtus: this portion was also attached to the child, by means of the funis, and was itself strongly adherent to the mesentery. The dilated intestine formed the most dependant part of the whole mass, and only covered a small portion of the posterior parts of the fœtus, the foot, fractured and bent carpus.

"It would be difficult to imagine, contrary to the influence of gravity, that, as the fœtus grew, it should ascend, and should make the ascent from the loose floating intestine, and in the direction of the funis from which it received its supply of blood, towards the higher, or fixed and immoveable part, the child's spine. Yet this must be the case upon the idea of the ovum which formed the fœtus having been deposited in the intestine. Again, the muscular fibres of the intestine not only formed the lowest part of the sac, but were to be traced into the superior, where they were lost; and about this part a considerable contraction of the cavity of the sac was observable.

"If we view it somewhat in the light of an extra-uterine fœtus, with the difference of a double conception; and that, by some accident, which it is not very difficult to imagine, the impregnated ova got connected together, the one forming an attachment to the uterus of the mother, and the other, (the fœtus in question,) to its twin twin brother; if, I say, this view of the subject be taken, there seems nothing in the matter which is wholly at variance with the known laws respecting generation."

In the last paragraph we cannot entirely agree with Mr. Highmore, unless he means by the word *wholly* to demand some reservation from the more obvious conclusion. The difficulty with us is as to the growth of the fœtus. If the boy, till the age of seven years, showed no preternatural bulk, we must recollect that when first born he must have concealed within his abdomen a substance larger than that which, at the age of seventeen, produced a visible tumour in that part of the abdomen under which it was situated. Now, what we wish to ask is as to the time of growth in this fœtus? If it was a twin thus preternaturally involved with its brother, its growth should have been whilst both were in utero, and then there would certainly have been nothing at variance with what we often meet with. But, if this fœtus grew after his brother left the womb, the fact is entirely new, and puts all our boasted knowledge of the fœtal state as to time and mode of existence, very much at nought. Mr. Highmore seems to concede that the fœtus grew in the abdomen of his adult brother, when he remarks the difficulty of supposing, as the fœtus grew, that it should ascend, *contrary to the influence of gravity*, a difficulty which could not have existed whilst the containing twin was in utero.

We might be led to suppose that the whole was nothing more than one of those unformed substances found most commonly in the ovaries of the human female, but met with also in other parts, consisting, for the most part, of teeth. But we are most of all surprised to find that no examination took place of the internal structure of this fœtus. Mr. Highmore, indeed, says, it was found necessary to *defer all such examination*. We may, therefore, hope, that some time or other it may yet take place. To conclude, much as we feel obliged to Mr. Highmore for his valuable communication, we cannot conceive the fact, even viewed as he would wish, as "coming within any of the known laws of generation," or more than slightly analogous with Mr. Young's.

---

*A View of the Mercurial Practice in Febrile Diseases*; by John Warren, M.D. President of the Massachusett's Medical Society, and Professor of Anatomy and Surgery in the University of Cambridge. 8vo. pp. 187. Boston, 1813.

It is always pleasing and sometimes useful to know the opinions, and be acquainted with the practice of our brethren in foreign countries. British practitioners, perhaps, have less to learn abroad than those of any other country, yet we often acquire the knowledge of new articles of materia medica, a more successful application of those with which we are already acquainted, and occasionally learn new principles from the works and communications of fellow labourers in various and remote parts of the world. The United States have furnished an ample list of scientific and zealous practitioners, amongst whom the present author deservedly ranks very high.

Dr. Warren commences with a general history of the practice, from which it appears, that it is only of late years that mercury has constituted the important part in the curative process of febrile diseases, which it now holds. The second section contains an account of the different preparations of mercury; the third, treats of the general operation of mercurials on the body; the fourth, on the operation of mercurials as stimulants; the fifth, on their operation on disease as stimulants; the sixth, on their effecting a change of action in the system; and the seventh, is upon salivation. We have not dwelt on this portion of the work, because the subjects are sufficiently familiar, and we do not observe any novelty in the facts that are adduced.

The author's plan consists in a fair exposition of facts, cases, and opinions, from various and copious authorities, together with his own experience. His statements are clear, and placed before the reader in such a strong light, that, if ever he is in the habit of reasoning, he cannot well be mistaken in the conclusions, which are most obvious and distinct.

Part II. Sect. 1. is on the general description of epidemic fevers. From the facts stated, it appears that there is a difference between inflammatory fever and typhus fever; that contagion is not peculiar to fevers, either of the inflammatory or of the typhoid form; but, when it does occur, which sometimes is the case, in either form, it is in some measure accidental, and depending on causes which have not been discovered.

The second section on the mercurial practice in epidemics is more important, as it brings us immediately into a very interesting inquiry. The yellow fever stands first on the list; we shall not now enumerate the symptoms, because they have been well related by several writers in our country, whose works are in every one's hands. But, as some difference of opinion has existed, and even continues to exist, respecting the cure of those fevers, it will be useful to give the result of what the author has collected on this subject, ject. At the same time we may remark, that some of the fevers described by the authors quoted by Dr. Warren, differ in several particulars from the disease described under the term Yellow Fever; some of them are termed inflammatory, some malignant pestilential, others yellow remitting fever, &c. &c. In fact, it is easy to see that the character of fevers, especially in the West Indies, is much influenced by circumstances.

Dr. Chisholm is one of the earliest practitioners who pushed mercury to any extent in the cure of these fevers, for, though it had been prescribed by several physicians before him, the practice was not in much repute; so lately even as 1789 and 1790, it was generally ridiculed in Jamaica and some other parts of the West Indies.

The following extracts contain ample illustrations in favour of the mercurial practice and is a fair specimen of the nature of Dr. Warren's publication.

"Dr. Chisholm having been led, as we have observed, to this practice, by his discovery in dissections, that the liver was principally diseased; and, by experience, that mercury was the sovereign medicine in hepatic complaints, confluent small-pox, and hydrocephalus internus, where the brain was compressed, after moderate evacuations, gave a pill, consisting of five grains of calomel, two of antimonial powder, and one of opium, eight times in twenty-four hours. In one case, four hundred grains were given before the salivary glands were affected.

"In 1794, from his former experience, he began the use of calomel without previous evacuations, and pursued it till salivation was induced; and did not lose a patient, in whom this practice was pushed to the full extent. Ten grains of calomel, with an equal or double quantity of jalap were, however, given usually for the first dose, which likewise evacuated the bowels in one or two hours, after which he gave ten grains every three hours, with or without opium, according to the effect on the bowels, till salivation was produced, which was generally in twenty-four hours.

"The action of the mercury was generally perceived after the third dose; the patient becoming calmer and less anxious; the skin softer, and of an agreeable heat; the stomach recovered its retentive faculty, and the patient was free from the disease. Salivation was supposed to be necessary, but immense quantities were required to effect it.

"The following are specimens of the symptoms and his methods of cure in the malignant pestilential fever of 1793, as exhibited in the practice of Dr. Campbell, who adopted his mode, and from these histories the reader must judge of the nature of the disease and its distinctive character.

"I. March 18, 1793. J——— was seized suddenly with violent head-ach, dimness of sight, a cold shivering, which was succeeded by convulsions. On recovering from these, he became extremely tremely hot, with severe pain in the legs and thighs. These symptoms, notwithstanding a profuse perspiration, continued without any abatement till the end of thirty-six hours. He was then very low, his pulse pretty full, but stopped by the slightest compression; his eyes and skin yellow, the latter without heat; his stomach incapable of retaining any thing. He had violent pain in the right side, a few spots of a dull purple colour on his breast and shoulders; his urine was small in quantity, of a pale yellow colour.

"On the 21st, he was ordered a pill of four grains of calomel, and half a grain of opium every four hours. 22d, symptoms the same; he vomited up some of the pills, but was ordered to continue them. 23d, kept down the pills. 24th, he was spitting plentifully. Every symptom disappeared.

"2. Mr. Taylor was seized with the usual symptoms, on the 9th, took the calomel and jalap as a purge; and on the 10th, began the pills as in the first case, with the addition of the antimonial powder. On the 12th, a ptyalism took place with relief from every complaint, excepting weakness.

"3. T. S. took, first, a purge of jalap and calomel on the 11th of June; on the 13th, six grains of calomel every four hours; on the 14th, eighteen grains of calomel with two of opium, twice in the day; 15th, the same quantity was repeated, mouth a little sore. The pulse then had become remarkably small and quick, but soft. 16th, pulse full and more natural, yellowness diminished. 17th, mouth very sore. 18th, salivated. Recovered.

"4. June 14th, R. M. began with the calomel, eight grains at a dose every eight hours; increased the quantity to forty and fifty grains a day, till at length, on the 22d, he took fifty-four, at which time he had taken 254 grains without affecting the mouth, and without its purging him. From this day the calomel was discontinued; but on the 24th, his mouth became sore. On the 25th, he was salivated, after which he began to recover.

"5. D. R. began with the calomel on the 25th of July, from which time to the 29th, he took 130 grains without affecting the mouth, and died the first of August.

"6. J. B. was seized on the 25th of July. On the 26th, he took five grains of calomel, with one grain and a half of opium. The calomel was repeated every three hours. 27th, quantity doubled. 28th, continual vomiting; coma and delirium ensued. Died in seventy-two hours from the attack. Blisters, bark, James's powders, and other auxiliary means were occasionally used in these cases; but, as no remarkable effects from those means were noted, they are not related in the above summary of the treatment. The pulse in these cases, when a ptyalism took place, was generally slower and softer.

"The following are subjoined as cases of the yellow remittent.*

"1. W. S.

* "As it is extremely difficult, if not impossible to determine, from the descriptions that have been published, the precise nature of

"1. W. S. aged 21, April 13, 1798, was seized with total insensibility; pulse indistinct, skin very warm; on being bled, the pulse instantly rose. 14th, pulse softer, still indistinct; insensibility continued.

"He took, in the course of the day, eighty grains of calomel. In the evening was more sensible. 15th, outrageously delirious; fifteen grains of calomel, with ten of camphor, and three of James's powder, were given every four hours; pulse 130, tongue whitish. 16th, had intervals of sensibility; belly open, skin cool; calomel continued in doses of ten grains. In the evening pulse ninety-two, skin cool, perfectly collected in his mind; pains across the fore-head; tongue covered with a thick white crust; eyes much inflamed. 17th, highly delirious, pulse 108, skin soft, and moist, tongue dry and brown in the middle, moist towards the edge; calomel continued. 18th, pulse 108, tongue moist; has had much sleep, is perfectly sensible, crust separating from the tongue. 19th, mouth sore; had rested well, has a return of appetite, and afterwards continued to recover.

"2. July 1, 1798, J. B. was seized with pains in his loins, across his eyes, and generally through the head, with some degree of heat, which continued through the 2d, when he took a purge of calomel with James's powder; after which he had a remission of symptoms; but, on the 3d, head-ach returning, he took thirty grains of calomel with two of opium in the course of the day. 4th, had an easy night; but in the morning, a violent return of pains; took pills with jalap and calomel, sixty grains of the latter, without any evident effect whatever. 5th, vomiting alarming, with black and ropy discharge like coffee-grounds; pulse 106, clammy sweats, without particular pains; pulse in the evening 116; continued the calomel. 6th, pulse 110, vomiting incessant; a mercurial clyster was injected every four hours, consisting of

Submur. Hydrargyri, ℥ss,

Tinct. Opii, ʒij.

Mucilaginis, q. s.

Aq. tepidæ, ℔ss.

And the parts on which blisters had been drawn were drest with the mercurial ointment.

"After the third clyster the vomiting ceased, the mouth became sore, and a gentle spitting came on, very troublesome hiccough ensued; pulse eighty-eight. 8th, Salivation was increasing with discharge of blood from the gums. 9th, This last circumstance continued, but the patient was somewhat better; and on the 12th, much better—recovered.

"In the case of J. W. ten grains of calomel were given with one

---

of the various epidemic fevers in the West Indies; and, as the mercurial practice was adopted in all of them, we shall not attempt very precisely to draw the line of distinction between them in the following statements."

grain of opium every four hours, from December 6th to 9th, when the patient was relieved, the mercury suspended, and angustura bark prescribed. On the 10th he relapsed and died. On dissection the membranes of the brain were found to be much inflamed; and much dark blood flowed on opening the cranium.

"Water was found in the ventricles, and also in the pericardium. The stomach was diminished in size, and the interior coat abraded. The liver was of the usual size, but of a cineritious colour. The gall bladder was tinged with excessively black bile; the spleen enlarged, and the inner surface of the intestines covered with pus.

"But the quantities of mercury exhibited in these cases are small in comparison with what were given in many others; and there seems to have been no other rule of prescription as to quantity, than to give it in proportion to the existing degree of torpor in the system, and till this was subdued.

"In one instance in Port Royal 2500 grains of mercury, in different ways, were used before the glands were affected.

"In 1799, W. G. took sixty-four grains by the mouth; 2040 by clyster; and thirty-six hundred grains of triturated mercury were carefully rubbed into his arms and thighs in five days, amounting in the whole to 5704 grains, and the cure was astonishingly rapid.

"1793, J. B. took at first twenty grains of calomel five times a day, and, when the case became apparently desperate, sixty grains at a dose till the 9th or 10th day, when, after having taken 800 grains, he was salivated and recovered.

In the fever at Tortola 1100 grains have been given by Dr. Porter, who lost one in seven, without salivation; and 1000 by Dr. Sanderson. At Jamaica, 1600 with advantage. But it has been observed, that the greatest success was insured, when the modes of application were varied. Instances have been adduced, wherein this disease, in a tropical climate, has resisted 2000 grains of calomel.

"The success of the mercurial practice in Grenada has been supposed to justify it; and, though it is a fact, that in the Royal Artillery, on whom it was first tried, the mortality was greater than had ever been known in a tropical climate; yet, compared with other modes of treatment, it was on the whole the most successful. About one in forty-eight, in whom the salivary glands were affected, died; and it was remarkable, that not one relapsed.

"We have the testimony of Dr. Lindsay to the comparative effects of this practice, in the army under Sir Charles Gray, in which it was largely used; and of that in the army under Sir Ralph Abercrombie, where it was not adopted, and the mortality was much less in the former.

"Mr. White in the same fever practised this method; and did not lose one in whom he could affect the salivary glands.

"About the same time, or a little before, the yellow fever commenced menced its ravages in Grenada, it appeared on board the Busbridge, East Indiaman.

"The treatment adopted by Mr. Bryce was commenced by first evacuating the contents of the bowels with mercurial cathartics These were frequently repeated, and from their favourable effect, compared with that of other cathartics, he was induced to suspect, that the disease was seated in the liver, pancreas, or spleen. Hence he concluded, that it was necessary to excite the action of the vessels; and from the known operation of mercury, he presumed it to be well adapted to that purpose. Mercurial frictions on the region of the liver were observed almost invariably to produce, soon after, one or more alvine evacuations of foul matter. Vomiting was the most troublesome attendant of this disease, the irritability of the stomach, in hot climates, being always extremely great. During the intervals of vomiting, calomel, given in large doses, had the happiest effect. All the excretions were promoted, particularly those of the liver, which was the principal seat of the complaint.

"It is remarkable, that about the same time, those two practitioners, Dr. Chisholm and Mr. Bryce, should have come to the same conclusion, with respect to this disease and its remedy, by different paths; the one, from the diseased state of the liver, and the other abdominal viscera, having inferred the aptitude of mercury for the cure of the disease; the other, without the help of dissections, from the success of mercury having deduced the nature of the complaint. This circumstance may, perhaps, with justice be urged as strong evidence in favour of the treatment. Three only out of three hundred are said to have died under Mr. Bryce's practice.

"In Trinidad, Dr. Clark observes, with respect to the fever which prevailed there in 1793, that, where there was time for salivating, mercury was always successful.

"In Dominica, the same fever was treated with mercurials, and Dr. Fillan asserts, that the proportion of mortality under it was about as 1 to 5, and as 1 to 2 under any other mode.

"Dr. Johnson makes the proportion who died under the same treatment as 1 to 7.

"In Grenada, Mr. Campbell, in 1796, lost about 1 in 7.

"In Antigua, Dr. Byam used mercury only on the decline of the epidemic; and the proportion of mortality was as 1 to 2.

"In St. Christophers, Dr. Armstrong in the same fever used mercury, and lost but 1 in 20. At the next period of its return, he aided the action of this medicine by the cold bath, and found this increased its activity. Dr. Noble declares, that when it affected the gums, the patient was secure; and that the cold bath was not incompatible with the process.*

"1796.

"* Mr. Muttleberry adopted this practice in 1796, on a battalion of grenadiers on-board the Valentine East Indiaman, and strongly testifies

"1796. In St. Lucia, where the yellow remittent fever prevailed more than in any of the other islands, calomel was given in doses of eight or ten grains, every three hours; and when the mouth was affected,* which was expedited by the cold bath, an amendment was observed. The mortality under this practice, by Dr. Allan, was as 1 to 7.

"In St. Vincents, the principal dependence of Dr. Davidson was on salivation.

"In St. Thomas's, this practice was recommended by Dr. Stevens; but the success was not great.

"The fever, which prevailed in St. Domingo, according to Dr. H. M'Lean, was not contagious; and differed essentially from that described by Drs. Chisholm and Rush.† He considers it as the common remittent, owing to chemical changes from miasmata promoting irregular determinations to particular organs, in consequence of diminished energy of the affected parts. An increase of the secretion of bile was a common attendant, and evident marks of inflammation in the stomach, and the black vomit, which is probably a mixture of the common juices of the stomach with blood, proceeding from the surcharged vessels.

"Though Dr. M. seldom used mercury but as a purge, and was of opinion, that it was too slow in its operation; and that, when it salivated, it was a dangerous circumstance in hot climates, because not easily controlled by medicine; yet, in bilious habits, which he always found difficult to correct, he used a slight mercurial course; and observed, that, when the system was fairly loaded, the symptoms abated.

"Dr. Todd gave 500 grs. to a woman in the same fever at Jamaica without any effect upon the mouth, and without success, even though a common cathartic operated on the bowels.

"In Demarara a fever of the usual character prevailed in 1796. The mercurial practice was adopted by Messrs. Macbeth and Ord, whilst the other practitioners used bleeding, antimonial evacuants, and peruvian bark. These latter almost universally failed, whilst the former as frequently succeeded;—and it is worthy of remark, that small quantities of mercury produced salivation, as it is said, in consequence of the moisture of the atmosphere.

"The Flora transport, in 1796, bound to the West Indies, had 23 of her crew taken sick with this fever. Mr. Ronaldson gave

---

testifies in favour of his success in comparison with other methods of treatment."

"* Dr. Peter and Dr. Sanderson found that the operation of mercury was surprisingly assisted by a few grains of Jalap."

"† Dr. Wade lost none in the treatment of what he calls the bilious fever out of forty-eight, whom he treated with mercury in Grenada in 1791; and Dr. Chisholm but one, provided the *salivary glands were affected*. And not a single relapse occurred. Vide Dr. Rush, p. 291."

10 grs.

10 grs. of calomel every three hours, and continued it, till their mouths were affected. In thirty-six hours 18 were convalescent. Of the whole crew, about 50 of whom were sick in their passage, he lost only three, whom he could not salivate, though their breaths were affected. It was observed, that those treated with mercury never relapsed, whilst those, who had been apparently cured by other means, often did; and were afterwards cured by mercury. One was a woman who was salivated during the catamenia without interruption to the curative process.

"At Port Royal, Dr. Davidson had an extraordinary case, in which calomel was given every three hours in doses of 10 grs. He began the treatment on the 22d of July; the mouth was affected in the evening, and the fever entirely gone off. The calomel was omitted. 23d, the soreness of the mouth went completely off, and the fever returned, with violent pains in the head, back, and legs. The mercury was administered again; in the evening, the mouth became sore, and he again desisted from the use of it. On the morning of the 24th, the mouth was well again, and all the symptoms returned. This obliged him to have recourse a third time to calomel, which was followed up, till the mouth was very much affected; and the patient recovered in a few days.

"Dr. Bishop, at the same time, lost only two patients out of forty-five, whom he salivated.—Salivation was always the signal of returning health."

Notwithstanding all this mass of evidence, and much more which we might have quoted, some physicians still object to the practice; but the arguments on which they ground their opposition are not very convincing, and Dr. Warren has not adduced many facts against the practice, although Dr. R. Jackson and Dr. Lind are amongst its antagonists. We believe mischief has been occasioned by pushing the remedy too far, so as to produce a cruel and needless salivation; but no bad consequences have ensued from the judicious employment of mercury.

In typhus fever, as it appeared at Boston, and differing from yellow fever, mercury also proved successful; it was not, however, pushed to the extent already noticed. In the winter of 1804 and 1805, the author describes a typhus which occurred in the town with uncommonly malignant symptoms, and under peculiar circumstances, in which from the cases adduced, mercury did not appear to us to have been copiously beneficial. Indeed, the deaths bore so great a proportion to the recoveries, that we doubt whether the mortality would have been greater had the cases been left to themselves. We have inserted some of these as a specimen of the complaint, as well as of the treatment.

"First Case. T. E. aged fifteen, employed in a store on Central Wharf, in the beginning of December, 1804, began to complain of slight pain in the head, languor and lassitude; and, being too unwell to continue his services in the store, returned to the house of his father, situated in Middle-street, near the head of Cross-street. The house is of brick, rather small, and with a northerly aspect. When visited by his physician, which was in the middle of the month, his countenance was remarkably pale, his appetite gone; he had nausea and slight vomiting; his tongue was covered with a thin whitish fur, pulse small and frequent; he had universal uneasiness, but was free from pains, excepting slight ones in the back and limbs. He had a constant chilliness, which made him unwilling to leave the fire a single moment, in consequence of which, he insensibly blistered his legs.—These symptoms were attended with restlessness in the night, which deprived him of sleep, and with obstinate constipation of the bowels.

" An emetic was administered, which operated kindly; and the next day an infusion of senna, which produced copious evacuations of a black and very offensive nature.

" On the day following, as the debility was extreme, and the surface of the body appeared destitute of colour and warmth, cinchona and snakeroot were liberally prescribed. Blisters were applied to the neck, temples, behind the ears, and on the extremities.—Wine, porter, and cyder, were freely allowed him. On the third of January, 1805, the course having been varied occasionally with ether, tinct. cinnam. and laudanum, he died, it being about the twenty-seventh day from his seizure.

" The spots, which had been blistered by the fire, before his death, assumed a purple hue; and others had appeared about his ears, but immediately subsided.

" Second. On the twenty-third of December, G. E. brother to the deceased, after five or six days labouring under the precursors of fever, became the subject of medical attention. He had slept with his sick brother, though efforts had been made to have him removed. He first complained of pains in the head, loins, and most of the muscles of the body, was alternately, at short intervals, cold and hot; had a nausea, and was costive; his tongue was covered with a dark fur in the middle, and a whitish mucus on the sides.

" He was ordered an emetic, which operated well, and afterwards a purge, which caused discharges of large quantities of black, putrid, and very offensive fœces. The next day he began with calomel in small doses, which soon affected the glands of the mouth. The fever, notwithstanding, ran through its various stages; the tongue became black and dry; he fell into a state of insensibility, and continued in it without speaking, for a number of days before the termination of his fever. Ether, laudanum, blisters to the back of the neck, behind the ears, on the temples, and the extremities, were the principal remedies used after he became insensible. Very little of nutriment or drinks could be forced into the stomach, till about the nineteenth or twentieth, when the fever left him, and he recovered his senses. He was soon soon after able to sit up in his chair; his appetite returned, and was, perhaps, indulged to his injury. He began to acquire flesh, till being carried below stairs, at his urgent request, to see his deceased brother, after five or six days convalescence, he relapsed. A colliquative diarrhœa having set in, and exhausted his strength to an alarming degree, it was corrected and restrained by rhubarb, tinct. of cinnamon, and laudanum. At this period of the complaint, I visited him with Dr. Rand, and found him extremely debilitated, and affected with a remarkable tumefaction on the right side of his face. On inspecting the inside of the mouth, an incipient mortification was discovered. The next day it had so far advanced, that he with great facility extracted the grinders of that side with his fingers; and it was remarkable, that no blood followed. At this time a small black spot began to shew itself on the outside of the right cheek; this in a few days extended itself over the whole of that cheek, which now became quite black; and all that side of the face became mortified from the inferior edge of the lower jaw to the orbit of the eye, and as far back as the ear.

"The eye was destroyed, and the face so distorted by the tumefaction on that side, that the countenance was a spectacle too shocking to behold. Liquids taken in by the mouth were discharged through the cheeks. He never lost his senses after the relapse, and took every day more than a bottle of Madeira wine, besides porter, brandy-punch, cider, and as much liquid nourishment as when well. The gums were sore from the calomel; but he expired on the twentieth of January, about the fourteenth day from the return of his disease.

"Third. E. E. was seized on the twentieth, with the same disease, under similar symptoms. Calomel in small doses was given him, and, as he could, neither by force nor persuasion, be induced to take any other, the case was trusted to this medicine alone, excepting the application of blisters to the nape of his neck and behind his ears; his mouth became sore, and he recovered, after a large abscess behind his right ear, which suppurated and healed kindly.

"Fourth. E. aged ten, was at about the same time taken sick. She was immediately put under the use of mercury; and being speedily removed into another house, rapidly recovered, after having taken only half a dozen pills, containing each one grain of calomel.

"Fifth. Eliza E. during the chief part of the illness of her brothers and sister, had laboured under leucorrhœa so profuse, as extremely to debilitate her; and had, in consequence, been confined part of the time to her bed in the same room with one of the sick; but, as she made no great complaint, she was not particularly attended to; and there was no suspicion of her having any other disease till at an advanced period, when she first became the object of medical attention.

"On the first examination were discovered, on her feet and legs, large petechiæ with elevations of the cuticle filled with a black sanious sanious fluid. The whole surface of her body was covered with vibices, and black stripes in various directions. Her pulse was very small and quick, with coldness of the extremities.

"In a few days she became delirious, screaming as if in extreme pain, but incapable of pointing out its seat. Her face early began to swell, and was of a cadaverous hue. The discharges from the bowels were black, putrid, and highly offensive. Swellings of the face and abdomen alternated with each other. She received very little nourishment, and scarcely a particle of medicine, it being impossible from her delirium and state of the stomach to get it down; and she died the last of January. The body became yellow after death, and putrefaction advanced with unusual rapidity."

Dr. Warren has given an interesting description of the yellow fever as it appeared in Boston in the year 1798. It did not materially differ from other fevers of the same class; and was not contagious. It occurred again in the year 1802, with all the circumstances of its former malignancy. It is curious and useful to trace the origin of such maladies, and, on this occasion, the author took great pains; yet, we are not entirely satisfied on the subject. It is of the first importance to ascertain whether the disease is imported, or whether it originates on the spot. The particulars which were ascertained on that occasion by the author's endeavours are these:

"The schooner Fair Attempt arrived at the quarantine ground on the 16th of June, in twenty days from Port au Prince.

"Before her departure, Mr. S. B. had been taken sick on shore; came on board and lodged one night, and then went on shore again.

"P. B. was seized seven or eight days before the vessel arrived, and recovered.

"No other person had been sick, till she had been out about fifteen days, excepting Capt. Crawford, who had been confined with the rheumatism.

"J. N. became sick after the schooner came on soundings. He died on board with black vomit.

"P. S. was taken sick the day before her arrival; and died on the third day from his seizure.

"During quarantine, according to the report of Dr. Welsh, the port physician, the utmost care was taken to have every part of the vessel thoroughly cleansed; every source of infection was supposed by him to have been searched out and expelled.

"On the 4th of July she was released from her quarantine, and came up to town, to the head of Tileston's Wharf.

"A number of articles, such as curious shells, corals, and sea-fans, were brought in the schooner. Mr. H. K. Hall went into the hold of the vessel for some of these articles. It has been stated, that some of the clothing on board had been delivered to Hannah Fessenden and Nancy Smith to wash, which they accordingly did; and and, that two persons, Mr. C. and Mr. D. (to whom some other articles had been committed, and by whom they were buried on the 10th of August,) complained of a very offensive smell from them. And a very intelligent gentleman, to whom I am indebted for much of my information on the subject, heard one of them say, two days before the seizure of Hannah Fessenden, that he had taken those clothes which were left with her, and buried them. But Mr. Anderson, the mate, informed me, that he had been a boarder at Fessenden's house before the voyage, and a lodger there after the arrival of the schooner; that, to his certain knowledge, Hannah did not wash any clothes that had been on board; that all those which were on board, were washed before, excepting what were contained in a chest, which was placed in a store in another place, and which was not opened till after the fever was over; and, that the clothes of those who died, were all thrown overboard. The clothes found at Fessenden's, and buried by Mr. Davenport, were Mr. Anderson's own clothes, some of them new, which had been left by him before be went the voyage, and had never been used out of Boston.

"It appears, that Mr. Hall, the first person seized in the town, was taken sick not till twenty-one days after the schooner's arrival at the wharf. Mr. D. was seized about twenty days after he buried the clothes. Kendall was taken immediately after conveying down Hall's bed from the top of the house. H. Fessenden was seized in the act of washing some clothes."

The evidence favours the supposition that the disease was imported; yet there are some difficulties attending this opinion. If the disease was communicated from the vessel, or any article that it contained, contagion is implied. But the author observes, contagious disease has not been known to exert its influence so slowly on the habit as to produce no tokens of indisposition within twenty days from the infection; as must have been the case if D. took it when he buried the clothes; also, opposed to this tardy infection, is the case of Kendall and of H. Fessenden, in whom the effects were instantaneously obvious, which seems very improbable. In 1798, the disease was satisfactorily proved to have been indigenous; and, in 1802, there appears to have been local causes sufficient to generate fever, viz. in the flats in the vicinity, a hot sun had been for some time exerting a very powerful influence, and promoting putrefaction in the large quantity of dead vegetable and animal matter which they retained. The case of Mrs. B. will suffice to prove, that the yellow fever is not very readily communicated from one individual to another. "I found her (says Dr. Warren,) labouring under all the symptoms of the most concentrated form of yellow fever, and saw her expiring under every mark of its most aggravated malignity. The house was filled filled with numbers of people, who had no suspicion of the nature of her complaint till within a few hours of her death; and yet not a person was infected by her." No physicians were attacked with the disease, and very few of the nurses, though several attended through every stage of the disease, performing all the offices of humanity and affection without the smallest degree of complaint. If, then, we conclude that the disease was communicated from the vessel, it is somewhat singular that it should not prove contagious on shore. Dr. Chisholm, on the other hand, in his letter to Dr. Haygarth, most strenuously supports the contagious nature of yellow fever, and asserts that the "latent period of infection varies from a few days to two months." We shall not dispute the learned doctor's statements, but it must be evident that the disease which he has described must be different from that described by Dr. Warren. In fact, the opinions of men high in the profession, and fully competent to judge, are so directly opposite on this important subject, that it is impossible to reconcile them without admitting that the disease, on some occasions, may prove contagious, whilst under different circumstances it is not so. Again, there is a difference of opinion respecting the causes of the disease; the contagionists contend, that it does not arise from putridity of animal and vegetable matters, nor from filth, nor the state of the atmosphere, but from contagion generated in human bodies and propagated by effluvia. The anti-contagionists, on the contrary, can readily trace it to the combined operation of heat and moisture on dead vegetable and animal matter, or filth in a confined situation when the ventilation is impeded. If the facts stated by both parties are correct, we think that there is evidence of the disease being produced by all these means. But we have still doubts on the subject, and are not disposed to commit ourselves by deciding where the advocates of either side do not appear sufficiently cool and disinterested in examining the question. The epidemic which has occasionally visited Gibraltar, and produced such dreadful consequences, was unquestionably the yellow fever; yet, the epidemic of Gibraltar has been traced to a similar origin with that of Boston, New York, and other places, viz. a crowded population exposed to the effluvia or exhalations which arise from the process of putrefaction in vegetable or animal matters, generated in a confined neighbourhood, with marshes and stagnant waters, in the sea-port towns. The disease must originate somewhere; and surely it is more rational to search for its source amongst these nuisances of native growth, than always to be looking abroad for some unfortunate ship, of which it sometimes happens that none of the crew have been diseased. In favour also of indigenous growth is the fact that the disease often occurs to a number of individuals at the same period of time, although they may not have visited the sick, or been in the way of infection, further than living in the same neighbourhood and being exposed to the same vitiated atmosphere. The disease could not spread so rapidly as has been the case on many occasions, if the infection was solely communicated by intercourse with the sick. Besides, it appears, that the medical attendants who live in a *good air*, at a distance from the infected neighbourhood, are, in general, exempt from the disease; they visit their patients with impunity, because they do not remain long enough in the vitiated atmosphere to become affected by it, and the disease being only infectious under certain circumstances of filth, or where several persons are confined in a small house or chamber without proper ventilation. We can readily conceive, that a person sleeping in a bed occupied the preceding night by a patient labouring under the malignant, pestilential, or yellow fever, may become infected, the sheets and bedding being impregnated with the exhalations from a highly diseased human body; but too much stress has been laid upon the circumstance, and too much alarm has been the result of such occurrences, which, indeed, ought to occasion greater cleanliness and precaution than appears to have been the case in the instances alluded to.

From all that has been advanced, perhaps, our readers will decide, that the nature of contagion is still but imperfectly understood. We solicit their assistance in contributing facts and cases to illustrate the inquiry more fully, for we think it a very important one. If the narrative with which we shall terminate this article be correct, it proves that a contagious principle may be buried for a great number of years, and yet prove active upon being disturbed. The village of Eyam was one of the last places in England visited by the plague in 1666, the year after that in which three thousand persons in London were said to have died of it in one night. Mr. Mompesson was the rector of Eyam. The plague was brought thither in patterns of cloth, sent from London to a tailor in the village. It raged with great violence, and swept away four-fifths of the inhabitants. Mr. Mompesson (the above named) writes, "In the summer of 1757, five labouring men, inhabitants of Eyam, were digging amongst the plague-graves on the heathy mountain above the village, to make potato-ground for a cottage which had been built there. They came to something which had the appearance of having once been linen. Conscious of its situation, tuation, they instantly buried it again; but, in a few days, they all sickened of a putrid fever, and three out of the five died. It *(the disease)* was so contagious, that the sick could procure no attendance out of their own family. The disease proved mortal to seventy persons of Eyam." We have copied this curious relation from Scott's edition of Miss Seward's poetical works, but should have been better satisfied if the particulars had been communicated by a medical practitioner. Credulity is abundant, and truth hard to discern, by minds prepossessed with a love for the marvellous.*

---

PETIT, SERRES, *and* GOGUYER, *on the Entero-Mesenteric Fever*. Paris, 1813.

WE have seen two works published in Paris, in the year 1813, on a disease which had proved very mortal in that city, and to which has been given the name of entero-mesenteric fever. The first of these works is the joint production of M. Petit, Physician in the Hotel-Dieu, and of M. Serres, also a physician, and holding some office in the same great hospital. The second is by M. S. J. Goguyer Laprugne, and is an inaugural dissertation, in which he comments on the opinions of Messieurs Petit and Serres.

The characteristics and the nature of this disease were at first obscure, until very carefully noticed and elucidated by dissection by Petit and Serres.

The persons affected with this disease were, for the most part, young men who had been badly nourished, and who had recently arrived in Paris from the country. Yet the disease was noticed in some cases of a different description; in females, in those who had lived on good food, in old men, in such as had always lived in Paris, and in some who had never resided there at all before their sickness; these last having been brought from the country to the hospital.

---

* Since writing the above, we have heard, with regret, that Dr. Warren died the 4th of last April. The extraordinary honours with which his funeral was solemnized, attest the high estimation in which he was regarded by his fellow citizens. A public eulogy was pronounced by Dr. Jackson; a sermon was preached on the following sunday; both of which have been printed. The body was attended to the vault by all the members of the Havard University, the fellows of Massachusetts Medical Society, the members of the Academy of Arts and Sciences, the officers and members of the Grand Lodge of Massachusetts, the members of the Humane Society, the reverend clergy, and a concourse of citizens. We presume his son, Dr. John Collins Warren, will succeed him in the professorship, being associated with him in the office in 1809.

The

The disease was noticed among persons of all temperaments; but it was most frequent and most severe among those who were feeble and worn down.

It occurred at all seasons, but was more frequently mortal when the weather was cold and damp, than when it was warm and dry.

The disease was marked in its early period by a sense of debility, by loss of appetite, by general uneasiness, by irregular paroxysms of heat, and, in most instances, by a diarrhœa, more or less urgent. After these symptoms had continued for some time gradually increasing, the patients became quite unable to attend to any labour, or to take care of themselves, and then they resorted to the Hotel-Dieu. It was thought that the disease progressed very slowly as long as it was left to itself; but, when any active medicine was administered, such as an emetic or a cathartic, the disease was aggravated and accelerated. The same effects were produced by excess in eating or drinking.

When patients presented themselves at the Hotel-Dieu before the more grave symptoms had developed themselves, they commonly exhibited the characteristics which follow:

"Their physiognomy had an expression of sinking and sadness; the eye was dull, the countenance discoloured and livid, especially about the lips and wings of the nose; they preferred lying on the back, and had a repugnance to motion; the skin was remarkable for its roughness and dryness; there was a torpor and inaction in the intellectual faculties, which were, however, correctly exercised when excited; their answers were slow, though intelligent; during the day they exhibited few or no febrile symptoms, but in the evening and night these were more fully developed, the paroxysms coming on gradually, without chills, and without sudden augmentation of heat, and accompanied by injection of the sclerotica, and most commonly by delirium; this last symptom, almost always slight, was suspended without much difficulty by addressing to the patient precise questions; the thirst was great, the teeth dry, the tongue superficially covered with a coat of a dark grey colour; the alvine evacuations were liquid, consisting of bile and serous fluid, varying as to frequency and quantity, and always insufficient to account for the general prostration of strength; the abdomen was supple, not at all puffed up, and without any, or with very little, pain, except on examination; but, when one pressed rather deeply at its lower part, on the right side, between the spine of the ilium and the umbilicus, the patient manifested by his cries the pain which he experienced; the suffering in this case was rendered evident, even without any voluntary expression of it, by the spasmodic retraction of the lips and ala nasi, and by the general expression of pain in his whole countenance."

This is a picture of the disease when in a moderate degree, and and in its most simple state. When patients applied for relief at this period, the disease commonly yielded without much difficulty, especially if the weather was dry and mild. But, when it did not, or when the disease had been more advanced before a proper treatment was adopted, it assumed an aspect vastly more serious.

"The expression of prostration and sadness was much more decided; the general complexion of the face was more sombre and earthy, the cheeks of a livid violet colour; the eye dull and sunken, and always injected; the drowsiness and delirium became continual; answers were obtained with more difficulty, although they still continued to be correct; the skin dry, rough, and sometimes covered with petechiæ; the subsultus tendinum was frequent; the febrile symptoms continued through the day, but were augmented at evening with the other distressing symptoms, and with these harassed the patient through the night; the pulses were frequent, feeble, and easily compressed; the teeth dry and slightly dark coloured; the tongue was covered with a brown superficial coat, which seemed almost as if pulverable, very rarely with a thick black crust; the thirst very great; the abdomen more painful to the touch, the pain being still in some cases limited to the inferior and right part without swelling, in other cases occupying a great extent, and accompanied by meteorism; the alvine evacuations serous, fetid in most instances, but not in all, occurring frequently; urine much diminished; and a tendency to gangrene in parts excoriated, whether the excoriation had been produced accidentally or by epispastics."

Under these circumstances the prognosis was unfavourable, although sometimes, especially when the weather was good, patients recovered from this state. In these fortunate cases there was noticed a general amelioration of the symptoms, particularly of the local symptoms, the pain and tumefaction of the abdomen; and, from the first appearance of convalescence, there was a return of a sharp appetite for food. With these symptoms of convalescence, there took place evacuations of two kinds, which were deemed critical. 1st. The urine became copious, depositing a greyish and powdery sediment. This symptom appeared and disappeared several times during the recovery of the patient. 2d. The skin became soft and moist, and was often covered with a warm and copious sweat. The restoration to health was observed to be much more rapid when this evacuation appeared.

In this way the subjects of this tedious disease were restored at length to perfect health; but the recovery was never rapid, commonly very slow, and was always liable to be interrupted by slight deviations in respect to diet and regimen. Exposure to a cold or damp atmosphere was particularly injurious.

In

In the fatal cases, and these were the most frequent when the symptoms last described had occurred, the bad symptoms went on increasing, with very slight remissions, and those of short duration. The patient sunk gradually and exhausted, after having exhibited the most disgusting symptoms; for the vital functions continued until the powers of life were perfectly worn out; the actions continuing in the trunk, when the extremities were already dead.

" *Appearances after Death.*—The subjects were commonly found, on examination, further advanced in putrefaction than bodies destroyed by other diseases and under similar circumstances in other respects.

" The brain, the lungs, and the heart, were found in the same state as usual after common *adynamic* (putrid) fever.

" On the first view, the abdomen seemed to be in a natural state. Tracing the alimentary canal from its origin, nothing remarkable was discovered before arriving at the middle of the ilium. There one began to perceive external spots of an oval form, of the colour of wine,* occupying that portion of the canal which is opposite to its attachment to the mysentery. The number and size of these spots increased in approaching the cœcum, and it was very rare to find any of them on the large intestines. On feeling the intestine, in the parts occupied by these spots, there was perceived a greater thickness than in other parts.

" On opening the alimentary canal, and examining its internal surface, nothing was discovered except at the parts corresponding with the spots already described. In these parts, the mucous membrane was found affected in a peculiar manner.† The spots were exactly defined at their circumference, and were formed by a slight tumefaction of the membrane, the adjacent parts retaining their natural appearance. These spots were much more strongly marked, were larger and attended with more swelling, and likewise were more numerous in the parts near the cœcum, than in the higher parts of the ileum. At the lower part, they were often seen an inch and a half, and sometimes two inches in length, projecting more than a line from the surrounding surface, and presenting a confused mass, so as to obstruct, in great measure, the cavity of the intestine. In these places there could not be discovered any *valvulæ conniventes;* at the expence or by the extension of which the diseased parts had been developed. The dark appearance which was noticed externally in the diseased parts, arose from the colour of these spots seen through the transparent peritoneum.

" Independently of this kind of alteration, there were often

* Probably of a reddish purple, the colour of common claret.

† M. M. Petit and Serres have given coloured plates to represent the diseased parts.

found found insulated pustules, which varied in number in different cases, and which were scattered here and there over the internal surface of the intestine. These, when examined with attention, seemed not to be different in their nature from the diseased spots before described. They were thought to represent the separate elements of the same spots.

"The state of the mesenteric glands corresponded most commonly to that of the mucous membrane of the intestine. Those which maintained relations with the spots least diseased had acquired some increase of size, but not much beyond that which they commonly have; their texture was firm, and they were tinged of a light rose-colour. But those which corresponded with the more diseased parts of the canal, had acquired a more considerable size, which sometimes equalled that of a nut:* externally they were of a bluish-red colour, but internally of a bright red, as if fully injected; and their proper texture was not easily discoverable.

"Such were the two extremes of that which we call the state of engorgement. That state had its intermediate degrees, of which it is easy to form a conception. There is one, which was frequently met with, in which the parenchymatous substance of the gland had acquired a perfect resemblance, in respect to colour and consistence, to the substance of the kidney.

"When the disease had been of long duration, the injury of the intestine and of the glands was not ordinarily limited to a state of engorgement. The parts were then found in some measure destroyed by ulceration. The diseased parts in the intestines were flattened, scarcely rising above the surrounding surface, and had acquired a colour more dark and more livid. Upon one or many points of their surface were found small round ulcers, of three to six lines in diameter, the bottom of which was sometimes covered with a thick, black, and sanious coating, and sometimes was quite clean, exposing to view the muscular fibres and the peritoneal membrane in an unaltered state.

"In the same cases the glands of the mysentery were found much less voluminous than in the state of engorgement. Externally they were almost black; and internally they presented to view sometimes a substance of a dull brown colour, in which there could not be traced any vestige of organization, sometimes a half-fluid matter of a dirty white colour, contained in the external membrane as in a shell, to which it adhered only in consequence of its viscidity.

"The different degrees of organic affection were frequently observed in the same subject; but, in all cases, the parts nearest the cœcum exhibited the greatest progress in the disease."

Such are the appearances occasioned by this disease, as

* The original does not say of what nut; but, by the plate accompanying the description, it would seem that the nut referred to must be about equal in size to that of our common walnuts.

observed observed during life, and after death, by M. M. Petit and Serres. It was not, however, in all cases so simple, for sometimes it was found in combination with affections of other parts, such as inflammation of the stomach, and inflammation of the peritoneum.

The account we have given of the disease in question is derived solely from the work of M. M. Petit and Serres. Their descriptions are supported by the detail of a number of cases.

In respect to the nature of the disease, and the best mode of treatment, those gentlemen entertain opinions which it is the whole object of the dissertation by Laprugne to controvert. It is maintained by Petit and Serres,—1st, that the entero-mesenteric fever is essentially and primitively adynamic (putrid), and that consequently the treatment should be tonic and stimulant; 2d, that the fever, or general affection, ought alone to receive the whole or the principal attention of the physician; 3d, that the inflammatory affection of the intestines, far from contra-indicating the use of tonics, requires the administration of them.

M. Goguyer-Laprugne controverts these opinions, and endeavours to prove,—1st, that this disease consists solely in an inflammation of the mucous membrane of the intestines; 2d, that the fever, or general affection, is entirely symptomatic, and does not consequently require any consideration in the treatment; 3d, that the inflammation in this case properly requires for its cure the use of mucilaginous liquids.

To proceed to the analysis of the arguments on these points would lead us too far, and would not, perhaps, be very useful. While the French doctors disagree respecting a disease under their own inspection, we shall not presume to decide their disputes on this side of the Atlantic.

If the disease is of a peculiar kind, and does not occur among us, yet the statement we have made may be useful, by leading the attention of the faculty to inflammatory affections within the abdomen; affections which are too often overlooked, not only when the inflammation is slow and obscurely marked, but also when it is acute and violent. If our brethren would open the bodies of those who die of "bilious cholic," and some other analogous diseases, they would often find reason to justify our remark.

We will add, that before reading the works which have been the subjects of this article, it had often been observed by us that inflammatory affections of the intestines are much more common in and near the right iliac region, than in any other part; and that, even when the disease extends over a large portion of the abdomen, it appears to centre and to be most most severe in that region. It is true, however, that these observations were oftener made in cases where the peritoneal coat was affected, than where the mucous coat was the seat of the disease.

The above work not having come to our hands, we are happy in presenting our readers with this critique on it from the *New-England Journal* of April last.

### MEDICAL AND PHILOSOPHICAL INTELLIGENCE.

